# Clinical Impact of Prostate-Specific Membrane Antigen Positron Emission Tomography (PSMA-PET) on Staging, Biochemical Recurrence Detection, and Treatment Decision-Making in Prostate Cancer

**DOI:** 10.7759/cureus.114815

**Published:** 2026-08-19

**Authors:** Saad Aad, Ribal Houmani, Jad Houmani, Christie Saad, Hagop Sassounian, Lamia Daouk, Carl Karam, Mohamad Houmani

**Affiliations:** 1 Faculty of Medicine and and Medical Sciences, University of Balamand, Koura, LBN; 2 Faculty of Health Sciences, University of Balamand, Koura, LBN; 3 Faculty of Medicine, American University of Beirut, Beirut, LBN; 4 Faculty of Medicine, Lebanese University, Beirut, LBN

**Keywords:** biochemical recurrence, disease staging, molecular imaging, oligometastatic disease, prostate cancer, psma-pet, radioligand therapy, treatment decision-making

## Abstract

Prostate-specific membrane antigen positron emission tomography (PSMA-PET) has transformed the imaging approach to prostate cancer (PCa) by enabling biologically targeted detection of disease beyond the capabilities of conventional anatomical imaging. This review examines the clinical impact of PSMA-PET in primary staging, detection of biochemical recurrence, and treatment decision-making, and discusses its integration into contemporary clinical practice. Compared with computed tomography (CT), magnetic resonance imaging (MRI), and bone scintigraphy, PSMA-PET demonstrates superior diagnostic accuracy for identifying nodal and distant metastatic disease, particularly in patients with intermediate- and high-risk tumors. In newly diagnosed disease, improved detection of small-volume metastases frequently alters treatment intent and guides the selection of local or systemic therapies. In the setting of biochemical recurrence, detection rates correlate strongly with prostate-specific antigen (PSA) levels, allowing earlier localization of recurrent disease and more individualized salvage strategies. PSMA-PET findings increasingly influence radiotherapy planning, selection of metastasis-directed therapies, and eligibility for targeted treatments. Major clinical guidelines now incorporate PSMA-PET into recommended staging and recurrence evaluation pathways. Despite these advances, important limitations remain, including false-positive findings, variability in interpretation, incomplete histologic confirmation, and limited evidence linking imaging-guided management changes to improved long-term outcomes. PSMA-PET represents a major advance in prostate cancer imaging by improving staging accuracy and enabling more personalized treatment strategies, while ongoing prospective studies aim to clarify its impact on survival and quality of care.

## Introduction and background

Prostate cancer (PCa) continues to be one of the most common malignancies in men and a major source of cancer-related morbidity and mortality [1]. Staging and early recurrence detection are key to maximizing outcomes, given the strong reliance of therapeutic strategies on disease extent and biological risk stratification. For many years, traditional imaging modalities such as computed tomography (CT), magnetic resonance imaging (MRI), and bone scintigraphy have been the backbone of staging algorithms [2]. However, these modalities are based predominantly on morphological assessment and have limited sensitivity for the detection of small-volume nodal or distant metastatic disease [3,4].

The advent of prostate-specific membrane antigen positron emission tomography (PSMA-PET) has transformed this approach. By targeting a transmembrane glycoprotein that is highly overexpressed in PCa cells, PSMA-PET enables molecular-level disease detection [5]. In patients with biochemical recurrence, ^18^F-PSMA-1007 PET/CT has demonstrated a patient-based sensitivity of 95% (95% confidence interval (CI), 91%-99%) and a detection rate of 95%, with positivity increasing at higher PSA levels [3]. Its applications range from primary staging in high-risk patients to the localization of biochemical recurrence (BCR) at low levels of prostate-specific antigen (PSA) [3]. Importantly, PSMA-PET not only enhances detection but also significantly affects treatment intent, intensity, and candidacy for metastasis-directed and radioligand therapies [6].

This review synthesizes the biological rationale for PSMA-PET, its diagnostic accuracy in primary staging and BCR, its role in treatment decision-making, guideline integration, current limitations, and future directions.

## Review

Methods 

A narrative literature review was conducted to identify peer-reviewed publications on the clinical impact of PSMA-PET in PCa staging, biochemical recurrence detection, and treatment decision-making. PubMed/MEDLINE, Scopus, and Google Scholar were searched between January 2008 and February 2026 using combinations of the following terms: “PSMA-PET,” “prostate-specific membrane antigen,” “prostate cancer,” “biochemical recurrence,” “primary staging,” “treatment decision-making,” “radioligand therapy,” “oligometastatic,” and “salvage radiotherapy.” Database-specific Boolean combinations of these terms were adapted to the indexing requirements of each database. Reference lists of identified articles and major society guidelines, including those from the European Association of Urology and National Comprehensive Cancer Network, were also screened for additional relevant publications.

Eligible publications included systematic reviews, meta-analyses, prospective and retrospective clinical studies, randomized controlled trials, and major guideline documents addressing the diagnostic performance, clinical impact, or therapeutic implications of PSMA-PET in PCa. Preference was given to publications from the last decade and to studies published in peer-reviewed indexed journals. Articles not available in English, conference abstracts without full data, and publications without direct relevance to PSMA-PET in PCa were excluded. Titles and abstracts were screened for relevance, followed by full-text assessment of potentially eligible publications. The synthesis of findings was qualitative and organized thematically around the review’s primary domains: biological rationale, primary staging, biochemical recurrence, therapeutic decision-making, guideline integration, limitations, and future directions.

As this was a narrative review, a formal Preferred Reporting Items for Systematic Reviews and Meta-Analyses (PRISMA)-style accounting of records identified and excluded was not performed. A formal risk-of-bias assessment was likewise not undertaken; this limitation was considered when interpreting findings from heterogeneous observational, prospective, and retrospective studies.

Biological rationale: why PSMA-PET is qualitatively different

Limitations of Conventional Imaging and the Need for Molecular Targeting

PCa is one of the most common cancers in men and is associated with substantial morbidity and mortality [1]. In the setting of BCR, metabolic PET tracers such as ^11^C- and ^18^F-choline have been investigated for disease localization and treatment planning [2]. However, conventional imaging methods such as CT and MRI have limited sensitivity for detecting prostate cancer lesions, particularly at sites of metastatic disease [3,4].

CT and MRI have limited sensitivity for detecting pelvic lymph-node metastases, particularly in small or morphologically inconspicuous lymph nodes [4,6]. Conventional imaging may also have difficulty distinguishing malignant from benign lymphadenopathy. Similarly, bone scintigraphy has limited specificity for detecting bone metastases in patients with PCa [6]. In these settings, PSMA-PET can provide more accurate characterization of disease extent [6]. By overcoming important limitations of conventional imaging, PSMA-targeted imaging has emerged as an important tool in the management of patients with PCa [3,6].

PSMA Biology and Molecular Basis for Imaging Superiority

In recent years, targeted imaging of PSMA - also known as glutamate carboxypeptidase II, N-acetyl-α-linked acidic dipeptidase I, or folate hydrolase - has opened a new chapter in the diagnostic management of patients with PCa. PSMA is a type II transmembrane glycoprotein that is strongly overexpressed in PCa cells, with expression generally increasing with tumor dedifferentiation and disease progression [3,5].

Although monoclonal antibodies such as J591 were initially developed to target PSMA, the discovery of urea-based small molecules capable of binding PSMA facilitated the development of highly specific PSMA-targeted radiotracers for PET imaging of PCa [5].

Quantitative assessment of PSMA uptake can provide information relevant to disease characterization and treatment planning. Imaging is essential prior to PSMA-directed therapy, such as radioligand therapy, to determine target expression. Quantitative imaging may also assist treatment planning and dosimetry; quantitative single-photon emission computed tomography/computed tomography (SPECT/CT), for example, has been evaluated for estimating salivary gland absorbed dose in patients undergoing ^177^Lu-PSMA-617 radioligand therapy [7].

Clinical Implications of PSMA Quantification and Target Expression

The clinical utility of PSMA-PET/CT at primary staging is highly dependent on pretreatment risk stratification, with maximal diagnostic yield observed in patients with unfavorable intermediate-risk or high-risk disease [8]. In these populations, PSMA-PET/CT detects nodal or metastatic disease in approximately 15% of patients with intermediate-risk and 31% of patients with high-risk disease [9,10]. A comprehensive systematic review and meta-analysis by Mazzone et al. confirmed the diagnostic value of PSMA-PET/CT for primary staging, particularly in intermediate- and high-risk cohorts, where detection of extraprostatic disease can directly inform treatment selection [10]. High-risk features including PSA greater than 20 ng/mL, Gleason score of 8 or higher, or clinical stage T3a or higher (extracapsular extension) are associated with a higher probability of metastatic disease and may increase the diagnostic yield of molecular imaging [11,12]. Emerging evidence further supports the utility of PSMA-PET/CT in refining risk classification within intermediate-risk subcategories, particularly among patients with unfavorable prognostic features [8,9].

Primary staging in newly diagnosed prostate cancer

What Does PSMA-PET Detect Better?

Pelvic nodal disease (regional lymph node involvement, N1): For pelvic nodal staging in newly diagnosed intermediate- to high-risk patients, head-to-head meta-analyses comparing PSMA-PET/CT with multiparametric MRI reveal patient-level sensitivities of 68%-76% for PSMA imaging versus 43%-60% for MRI, with comparable specificities exceeding 90% across both modalities [13,14].

Occult distant metastases (distant metastatic disease, M1): Beyond locoregional staging, PSMA-PET/CT demonstrates improved detection of distant metastatic disease, particularly bone metastases that may be missed by conventional imaging. In the prospective Primary Distant Metastasis Staging of Prostate Cancer (PROSTAGE) study, ^18^F-PSMA-1007 PET/CT demonstrated high diagnostic performance for bone metastases, with an area under the curve (AUC) of 0.90 (95% CI, 0.85-0.95) and 0.91 (95% CI, 0.87-0.96) for two independent readers, and identified metastatic disease in 11 of 20 patients whose standard imaging was negative [15]. Similarly, a head-to-head comparison of ^68^Ga-PSMA-11 and ^18^F-PSMA-1007 PET/CT using histopathology and immunohistochemistry as reference standards demonstrated high diagnostic performance for primary staging [16]. In a systematic review and meta-analysis of head-to-head comparisons, PSMA-PET demonstrated significantly higher sensitivity and specificity than conventional imaging for bone metastasis detection, with pooled sensitivity of 98.0% and specificity of 96.2%, compared with 73.0% and 79.1%, respectively, for conventional imaging [17]. These findings support the role of PSMA-PET/CT in identifying occult distant disease and refining initial staging and treatment planning [15-17] (Table 1).

**Table 1:** Comparative diagnostic performance of PSMA-PET/CT versus conventional imaging in primary staging of intermediate- to high-risk prostate cancer. NPV: negative predictive value; PLND: pelvic lymph node dissection; mpMRI: multiparametric MRI; RT: radiotherapy; PSMA-PET: prostate-specific membrane antigen positron emission tomography; PCa: prostate cancer; CT: computed tomography; WB-MRI: whole-body MRI; SPECT-CT: single-photon emission computed tomography/computed tomography.

Study (Year)	Population	Comparator	Pelvic nodal sensitivity	Pelvic nodal specificity	Detection of occult M1 disease	Key clinical impact
Kuten et al. (2020) [16]	Newly diagnosed PCa	Histopathology+immunohistochemistry	High diagnostic performance	High specificity	Limited	Histopathologic validation of PSMA-PET/CT for primary staging
Pienta et al. (2021) [14]	Intermediate- to high-risk PCa	Histopathology	High sensitivity for pelvic nodal disease	High specificity	Detection of extrapelvic disease	Supported PSMA-PET/CT for initial staging
Anttinen et al. (2021) [15]	Newly diagnosed PCa	Conventional imaging/WB-MRI/SPECT/CT	Not primary endpoint	Not primary endpoint	Improved detection of distant metastases	Improved primary distant-metastasis staging
Hernes et al. (2021) [18]	Primary and recurrent PCa	Histopathology	Meta-analytic diagnostic accuracy	Meta-analytic diagnostic accuracy	-	Systematically evaluated PSMA-PET diagnostic accuracy using histopathology as reference
Wang et al. (2022) [13]	Intermediate–high-risk PCa / staging populations	WB-MRI/conventional imaging	Not primary endpoint	Not primary endpoint	Discussed improved detection of nodal and metastatic disease	Reviewed the emerging role of PSMA-PET and WB-MRI in next-generation staging
Chow et al. (2023) [17]	Intermediate–high-risk PCa	Conventional imaging	Higher sensitivity vs conventional imaging	Superior diagnostic accuracy vs conventional imaging	Higher detection of metastatic disease	Improved initial staging and treatment planning
Nikitas et al. (2024) [19]	Pretreatment and post-RP PCa	Decipher genomic risk	Not primary focus	Not primary focus	Nonlocalized disease (miN1M0 or miM1a-c) detected on PSMA-PET/CT	Higher Decipher scores associated with PSMA-PET–detected nonlocalized disease

Impact on Initial Treatment Intent

*Upstaging*: Avoidance of futile local-only therapy. PSMA-PET/CT can substantially alter treatment selection by identifying previously occult nodal or distant metastatic disease [20,21]. A systematic review and meta-analysis reported management changes in 28.0% (95% CI, 23.0%-34.0%) of patients undergoing primary staging and 54.0% (95% CI, 50.0%-58.0%) of patients evaluated for recurrent disease [22,23]. When upstaging reveals previously occult nodal or distant metastatic disease, PSMA-PET/CT can help avoid potentially futile local-only treatment and guide treatment toward systemic or metastasis-directed approaches [24,25]. For patients with initially localized high-risk disease, identification of low-volume M1 disease on pretreatment PSMA-PET/CT can alter treatment intent and redirect management toward systemic and/or metastasis-directed approaches [26]. In selected patients with limited metastatic disease, metastasis-directed stereotactic body radiotherapy (SBRT) represents a potential local treatment approach, with evidence supporting the use of hypofractionated treatment schedules [27].

*Downstaging*: Conversely, when PSMA-PET/CT yields negative findings in patients previously classified as high-risk by conventional imaging, appropriate downstaging can preserve curative-intent local therapies that might otherwise be withheld [28]. The extent of stage migration varies according to baseline disease risk and imaging findings, with prospective data demonstrating that PSMA-PET/CT can substantially reclassify patients compared with conventional imaging [28]. Negative PSMA-PET/CT may also help identify selected patients in whom extended pelvic lymph node dissection (PLND) could potentially be omitted; however, this approach remains dependent on baseline risk and the diagnostic performance of PSMA-PET/CT [20].

Biochemical recurrence

BCR after definitive therapy for PCa, defined as a rising PSA following curative therapy such as surgical resection of the prostate or radiotherapy, occurs in a substantial proportion of patients and represents a clinical challenge in current practice [29,30]. PSMA-PET has emerged as a novel imaging technique allowing earlier, more sensitive, and more precise detection of recurrent disease, directly influencing clinical management and salvage therapy decisions [31].

Detection Performance by PSA Level

PSMA-PET imaging demonstrates a clear correlation between detection rate and PSA level, a relationship confirmed across prospective studies and meta-analyses. Wu et al. conducted a meta-analysis of prospective studies evaluating different PSMA ligands, reporting that detection rates increased progressively with PSA [32]. Detection was lower at PSA below 0.2 ng/mL, rose significantly at PSA 0.2-0.5 ng/mL, increased further at PSA 0.5-1.0 ng/mL, and was highest at PSA above 1.0 ng/mL.

Evidence from prospective multicenter trials supports these findings. Fendler et al. assessed gallium-68 PSMA-11 PET in recurrent PCa and reported high sensitivity even at PSA levels below 0.5 ng/mL, noting that the detection probability increased steadily with rising PSA [33]. A similar observation was made by Meredith et al., who supported the idea that gallium-68 PSMA-PET/CT could detect recurrence in patients with very low PSA levels, enabling early salvage strategies that would have been missed by conventional imaging [34].

Notably, a German multicenter study focused on patients with PSA ≤0.2 ng/mL, comparing conventional tracers (gallium-68 PSMA-11, gallium-68 prostate-specific membrane antigen inhibitor and targeting (PSMA I&T), and fluorine-18 PSMA-1007), found an overall detection rate of approximately 30% even at these low PSA levels, demonstrating the value of PSMA-PET in identifying recurrence early in the disease course [35]. These low-level detections further emphasize its potential role in guiding early salvage therapy and preventing disease progression.

Patterns of Disease Uncovered

At low PSA levels, recurrence is often confined locally, allowing targeted salvage radiotherapy rather than empiric irradiation of larger fields [35]. As PSA rises, pelvic nodal involvement becomes increasingly prevalent, highlighting the need to adapt radiotherapy fields to include involved nodes that may otherwise go untreated with conventional imaging [36,37].

The spatial patterning of recurrence also influences the choice of radiotherapy technique. PSMA-PET allows for dose escalation to PSMA-avid lesions while sparing uninvolved tissue, reducing toxicity. In many patients, detection of isolated pelvic nodal or local recurrence enables field reduction, minimizing radiation exposure to adjacent organs [30]. Conversely, when PSMA-PET identifies additional disease outside the expected radiation field, clinicians may expand fields to ensure comprehensive coverage [35,37].

Impact on Salvage Radiotherapy

The influence of PSMA-PET on salvage radiotherapy planning is substantial. PSMA-PET allows clinicians to implement image-guided, individualized salvage strategies, optimizing both efficacy and treatment selection. In the CONDOR trial, ^18^F-DCFPyL (piflufolastat F 18, also known as PyL) PET/CT prompted a change in intended management in nearly two-thirds of patients with BCR, including modification of radiation fields and selection of metastasis-directed therapies [30,36]. Although these findings were obtained using ^18^F-DCFPyL, the management implications may not be directly generalizable across all PSMA-targeted tracers.

PSMA-PET can also identify patients who may not benefit from local salvage therapy alone due to occult systemic spread, enabling early consideration of systemic agents such as androgen deprivation therapy (ADT) or novel anti-androgens [33,35].

Emerging evidence further supports the prognostic utility of PSMA-PET beyond detection. Kudura et al. showed that volumetric tumor segmentation metrics on PET correlate with survival outcomes, suggesting that patients with higher tumor burden may require more aggressive treatment or combined-modality approaches [38].

PSMA-PET as a determinant of therapeutic intent and intensity

Contemporary impact-focused reviews consistently demonstrate that PSMA-PET findings lead to management changes in a substantial proportion of patients, spanning newly diagnosed high-risk disease, BCR, and oligometastatic presentations [23,39,40]. These effects can be conceptualized across three major domains: preservation of curative intent, abandonment of curative intent when warranted, and treatment intensification or de-intensification.

Preservation of Curative Intent

In the recurrent setting, PSMA-PET allows radiation oncologists to transition from empiric pelvic salvage radiotherapy toward image-guided, lesion-directed strategies. Identification of discrete nodal or local recurrences permits focal dose escalation to PSMA-avid sites, selective pelvic nodal coverage, and avoidance of unnecessary irradiation in PET-negative regions. Dragonetti et al. emphasized that PSMA-guided radiotherapy improves target selection precision and enables biologically adapted dose intensification while potentially sparing uninvolved tissues [39]. Similarly, Weiner et al. reported that PSMA findings frequently lead to modification of radiation fields, often narrowing or expanding coverage depending on detected disease distribution [23].

The emergence of PSMA-PET has been particularly transformative in oligometastatic prostate cancer. Sabbagh et al. described how PSMA imaging refines the oligometastatic paradigm by detecting limited-volume metastatic disease earlier, enabling stereotactic body radiotherapy (SBRT) to individual lesions, and supporting metastasis-directed therapy as a strategy to delay systemic treatment [40]. By identifying patients with truly limited metastatic burden, PSMA-PET helps preserve curative or metastasis-directed approaches that might otherwise have been precluded under conventional imaging paradigms.

Abandonment of Curative Intent When Warranted

Equally important is the role of PSMA-PET in preventing futile local therapy. Unexpected M1 disease identified at initial staging or recurrence can shift management from definitive local therapy (e.g., radical prostatectomy or prostate-only radiotherapy) to systemic therapy (e.g., ADT with or without novel hormonal agents), or to systemic therapy combined with selected local control. Weiner et al. highlighted that upstaging to metastatic disease frequently alters intent from curative to systemic management, thus avoiding non-beneficial surgery or isolated pelvic radiotherapy in patients with occult distant spread [23].

Treatment Intensification and De-Intensification

PSMA-PET also influences therapeutic intensity within a given stage category. Intensification strategies include the addition of ADT to radiotherapy when nodal disease is identified, expansion of radiotherapy fields to include PSMA-positive nodal basins, and integration of systemic therapy in previously presumed localized disease. De-intensification strategies, by contrast, include the omission of elective pelvic radiotherapy in PET-negative patients, avoidance of unnecessary ADT when nodal involvement is not confirmed, and preservation of localized curative treatment in patients downstaged by PSMA imaging. Dragonetti et al. and Weiner et al. both underscored that PSMA-guided modifications often refine therapy in both directions, intensifying when biologically justified and de-intensifying when disease burden is lower than anticipated [23,39].

In metastatic castration-resistant prostate cancer (mCRPC), PSMA-PET/CT also contributes to the selection of patients for ^177^Lu-PSMA-617 radioligand therapy. The phase III VIsion Investigation of PSMA-Targeted Therapy in Metastatic Castration-Resistant Prostate Cancer (VISION) trial and the TheraP trial demonstrated clinical benefit of ^177^Lu-PSMA-617 in appropriately selected patients with PSMA-positive mCRPC. PSMA-PET-based assessment of tumor uptake can help characterize PSMA expression and support treatment selection, although specific uptake thresholds may vary according to the imaging protocol and clinical setting [39].

Stage Migration Versus Survival Benefit

A critical distinction must be emphasized: management change does not equate to proven survival benefit. While PSMA-PET consistently alters clinical decision-making, long-term overall survival (OS) and metastasis-free survival (MFS) data directly attributable to PSMA-guided strategies remain maturing. As discussed by Weiner et al., much of the current evidence demonstrates high rates of management modification, improved staging accuracy, and biologic plausibility for improved outcomes [23]. However, definitive randomized evidence demonstrating that PSMA-PET-guided management improves OS or MFS remains limited. The potential for stage migration (the “Will Rogers phenomenon”) must therefore be acknowledged: by detecting disease earlier and more sensitively, PSMA-PET may reclassify patients without necessarily altering the underlying biology or natural history of the disease. Importantly, ongoing prospective studies are increasingly linking PSMA-PET findings with clinical outcomes rather than management changes alone. These studies are evaluating endpoints such as biochemical progression, metastasis-free survival, treatment response, and overall survival, with the goal of determining whether PSMA-PET-guided treatment strategies translate into meaningful patient benefit. Until mature outcome data become available, the principal evidence supporting PSMA-PET remains its superior disease characterization and its ability to refine treatment selection, rather than a definitively established survival advantage [39,40].

Current guideline integration

From experimental modality to standard-of-care alternative, major international guidelines now recognize PSMA-PET as central to PCa imaging. The European Association of Urology (EAU) guidelines now recommend PSMA-PET/CT for primary staging of high-risk PCa when available and for evaluation of biochemical recurrence, particularly at low PSA levels. The National Comprehensive Cancer Network (NCCN) guidelines have integrated PSMA-PET into initial staging pathways for unfavorable intermediate- and high-risk disease and into BCR algorithms following radical prostatectomy or radiation therapy. PSMA-based imaging is accepted as an alternative - and in many scenarios as preferred - advanced imaging over conventional CT and bone scan (Table 2). The transition from emerging technology to standard-of-care alternative underscores the strength of the accumulated evidence demonstrating improved staging accuracy and clinically meaningful management impact.

**Table 2:** Guideline-of-guidelines synthesis: consensus recommendations for PSMA-PET use. Synthesis based on European Association of Urology (EAU) [9], National Comprehensive Cancer Network (NCCN), Advanced Prostate Cancer Consensus Conference (APCCC) recommendations [9,23,41]. PSMA-PET: Prostate-specific membrane antigen positron emission tomography; BCR: biochemical recurrence; PSA: prostate-specific antigen.

Clinical context	Consensus position across major guidelines	Practical interpretation
High-risk primary staging	PSMA-PET is appropriate and frequently preferred when available.	Use PSMA-PET upfront to improve detection of nodal and occult metastatic disease and guide initial treatment intent.
Biochemical recurrence	PSMA-PET is recommended due to superior detection at low PSA levels.	Use PSMA-PET early in BCR to localize recurrence and tailor salvage radiotherapy or metastasis-directed therapy.
Low-risk disease	Routine PSMA imaging is not recommended given limited incremental value.	Avoid routine PSMA-PET in low-risk patients because it rarely changes management and may increase incidental findings.
Clinical decision integration	Imaging findings should directly inform local, systemic, and metastasis-directed strategies.	Use PSMA-PET results to adjust intent (curative vs systemic), intensity (escalate or de-escalate), and targets (fields or lesions).

Proposed clinical algorithms for PSMA-PET integration

Imaging Algorithm for Primary Staging

PSMA-PET should be integrated into primary staging according to pretreatment risk stratification. In patients with unfavorable intermediate-risk or high-risk PCa, PSMA-PET is recommended to refine disease extent and guide treatment intent. When imaging confirms localized disease without nodal or distant metastases, curative local therapy remains appropriate, including radical prostatectomy with or without PLND, or definitive radiotherapy with ADT as indicated.

If pelvic nodal disease (N1) is identified, treatment intensification is warranted, typically consisting of extended-field radiotherapy combined with ADT, with consideration of multimodal approaches. In contrast, detection of distant metastatic disease (M1) shifts management toward a systemic-based strategy, incorporating ADT with or without novel hormonal agents. In selected oligometastatic cases, metastasis-directed therapy and local control strategies may be considered within a multidisciplinary framework.

Guideline-Based Imaging Algorithm for Biochemical Recurrence

In the setting of BCR, PSMA-PET is recommended following the confirmation of rising PSA after definitive therapy. After radical prostatectomy, biochemical recurrence is typically defined as a PSA level of ≥0.2 ng/mL confirmed by a second measurement, whereas after radiotherapy, BCR is generally defined according to the Phoenix criterion as a PSA level of 2.0 ng/mL above the post-treatment nadir. Given the strong correlation between detection rates and PSA levels, PSMA-PET should be considered early in the recurrence course to localize disease and guide salvage management.

Management is directed according to the pattern of recurrence identified. Local recurrence confined to the prostate bed supports salvage radiotherapy. Detection of pelvic nodal disease warrants targeted nodal radiotherapy with potential field expansion and ADT. In cases of oligometastatic disease (commonly defined as ≤5 lesions), metastasis-directed therapy such as SBRT or surgical resection may be considered, with or without systemic therapy. Widespread metastatic disease generally necessitates systemic treatment, including ADT and androgen receptor-targeted agents, with consideration of clinical trial enrollment.

Limitations and controversies

False-Positives and Imaging Pitfalls

Despite high diagnostic accuracy, PSMA-PET/CT is susceptible to false-positive findings that may lead to overstaging and inappropriate therapeutic escalation [41,42]. Physiologic PSMA expression occurs in sympathetic ganglia, salivary glands, kidneys, and small bowel, occasionally mimicking pathologic uptake and complicating interpretation [41,43]. Benign osseous uptake patterns, including nonspecific bone uptake (NSBU), healing fractures, degenerative changes, and Paget disease, represent frequent sources of false-positive results, particularly in elderly patients with concurrent skeletal pathology [44,45]. Recent systematic reviews have further characterized the distribution and patterns of nonspecific bone uptake associated with PSMA-targeted tracers [45]. Interobserver agreement in image interpretation is also relevant: a systematic review and meta-analysis of inter-observer variability in PSMA-PET interpretation reported substantial pooled agreement across lesion categories (κ between 0.60 and 0.80), with the highest agreement for regional lymphadenopathy (0.74) and the lowest for soft-tissue metastasis (0.65) [46].

Reader Variability and Interpretive Challenges

Inter-observer variability represents a persistent limitation in PSMA-PET/CT interpretation, with discrepancies arising in lesion detection, localization, and classification - particularly for small lymph nodes and equivocal skeletal lesions [46]. A systematic review and meta-analysis of observer agreement demonstrated moderate-to-substantial concordance for disease detection but highlighted inconsistencies in per-lesion analysis, especially in distinguishing physiologic uptake from pathology in borderline cases [46]. Variation in quantitative and qualitative imaging parameters, including standardized uptake value (SUV)-based measurements and PSMA uptake assessment, may contribute to heterogeneity in image interpretation and clinical assessment [47,48].

Lack of Universal Histologic Confirmation

A fundamental limitation of PSMA-PET/CT imaging is the frequent absence of histopathologic validation for lesions detected on imaging, particularly in advanced disease settings where biopsy is often clinically impractical or deferred [12,22].

Access, Cost, and Tracer Variability

Widespread implementation of PSMA-PET/CT remains constrained by limited access to specialized nuclear medicine facilities and radiopharmaceutical production infrastructure, particularly affecting rural and resource-limited settings [6,49]. Gallium-68-labeled tracers require on-site generator systems and exhibit short half-life constraints limiting geographic distribution, whereas fluorine-18-based agents offer extended shelf-life and centralized production capabilities that improve accessibility [50]. Comparative studies demonstrate that gallium-68 PSMA-11 and fluorine-18-based radiotracers exhibit comparable diagnostic accuracy but differ in biodistribution patterns, urinary excretion, and optimal imaging timepoints [50]. Certain ^18^F-labeled PSMA tracers, such as ^18^F-PSMA-1007, demonstrate lower urinary excretion than ^68^Ga-PSMA-11, potentially improving the visualization of pelvic lesions and local recurrence [36]. The lack of standardized protocols for tracer selection, administered activity, imaging timing, and reconstruction parameters complicates cross-institutional comparisons and introduces technical heterogeneity that may influence diagnostic outcomes [31].

Outcome Data Lag Behind Detection Data

Although PSMA-PET/CT demonstrates superior detection capabilities for PCa staging and recurrence identification, robust evidence linking improved imaging detection to tangible improvements in overall survival or cancer-specific mortality remains limited [23,51]. Most validation studies employ diagnostic accuracy endpoints rather than patient-centered outcomes, creating uncertainty about whether earlier disease detection translates into meaningful therapeutic benefit or simply advances diagnosis without altering ultimate prognosis [13,29]. While PSMA-PET/CT frequently redirects clinical management decisions - including treatment intensification or de-escalation - evidence from prospective randomized trials demonstrating survival advantages associated with PSMA-PET-guided therapy modifications is still emerging [37,52]. This evidence gap raises concerns about potential lead-time bias and overtreatment resulting from the detection of indolent disease that may not require immediate intervention [53]. Lead-time bias occurs when earlier detection through more sensitive imaging makes survival from the time of diagnosis appear longer without actually delaying death or altering the underlying course of disease; with PSMA-PET, earlier identification of otherwise occult disease may therefore improve measured survival from diagnosis without necessarily improving overall survival.

Future directions

Trials Linking PSMA-PET-Guided Management to Progression-Free Survival, Metastasis-Free Survival, and Quality of Life

While PSMA-PET/CT has demonstrated superior diagnostic accuracy, prospective randomized trials establishing its impact on patient-centered outcomes remain limited. Ongoing trials evaluating PSMA-PET-guided salvage radiotherapy, metastasis-directed therapy, and treatment intensification in oligometastatic disease are critical to validating clinical benefit beyond diagnostic performance alone [23,39].

Standardized Reporting Frameworks (PROMISE)

The rapid expansion of PSMA-PET/CT utilization has created substantial heterogeneity in image acquisition, interpretation, and reporting across institutions, threatening reproducibility and inter-trial comparability [54]. The Prostate Cancer Molecular Imaging Standardized Evaluation (PROMISE) framework was developed through international consensus to address these limitations, establishing uniform criteria for lesion characterization, anatomic localization, and quantitative assessment [54]. Complementary structured reporting systems such as Prostate-Specific Membrane Antigen Reporting and Data System (PSMA-RADS) provide diagnostic frameworks for clinical staging, enhancing communication between nuclear medicine physicians and multidisciplinary oncology teams [48]. Implementation of standardized imaging and biomarker assessment frameworks in pivotal trials such as TheraP, VISION, and ENZA-p has supported robust biomarker analyses and contributed to the evidence base underlying PSMA-targeted therapeutic development [55-57]. Future iterations of PROMISE are expected to integrate artificial intelligence-derived quantitative metrics and radiomics signatures, further refining prognostic stratification and treatment response prediction [54,58].

Integration With Radiomics and Artificial Intelligence

Artificial intelligence (AI) and radiomics represent transformative technologies for enhancing PSMA-PET/CT interpretation and prognostic stratification. Automated lesion detection algorithms reduce inter-observer variability and accelerate workflow efficiency, addressing a persistent challenge in high-volume clinical practice [59,60]. Deep-learning models demonstrate promising performance in distinguishing malignant PSMA-avid lesions from physiologic uptake patterns, minimizing false-positive interpretations that complicate treatment planning [59]. Radiomics-based feature extraction from PSMA-PET enables quantitative characterization of tumor heterogeneity, with specific signatures correlating with aggressive histopathology, higher Gleason scores, and adverse pathologic outcomes [61]. Machine learning models incorporating baseline PSMA-PET radiomic parameters show utility in predicting treatment response to radioligand therapy, ADT, and metastasis-directed interventions, facilitating personalized risk stratification [61]. Despite these advances, clinical implementation requires standardization of feature extraction methodologies, external validation across diverse populations, and regulatory approval through prospective multicenter trials [60,62]. Integration of AI-derived metrics into standardized reporting frameworks such as PROMISE represents a critical next step toward routine clinical adoption [63].

Harmonization of Treatment Pathways Based on PSMA Findings

The integration of PSMA-PET/CT into standardized treatment algorithms requires multidisciplinary consensus to optimize patient selection for curative, metastasis-directed, and systemic therapies [23]. International guidelines from the European Association of Urology-European Association of Nuclear Medicine (EAU-EANM) and the Advanced Prostate Cancer Consensus Conference (APCCC) increasingly incorporate PSMA-PET findings into decision-making frameworks for oligometastatic disease management, salvage radiation field design, and eligibility for lutetium-177 PSMA-617 radioligand therapy [39,40]. PSMA-defined oligometastatic PCa represents a therapeutic paradigm in which imaging increasingly influences treatment selection, with metastasis-directed therapies often guided by PSMA-PET lesion localization [21,64]. Harmonized reporting using the PROMISE criteria facilitates consistent communication between radiologists, urologists, radiation oncologists, and medical oncologists, enabling coordinated care pathways across institutions [48,54]. Despite growing adoption, variability persists in PSMA-PET access, reimbursement policies, and institutional protocols across different healthcare systems [65].

## Conclusions

PSMA-PET has reshaped PCa imaging by enabling biologically guided disease characterization rather than relying solely on morphological assessment. In both newly diagnosed and recurrent disease, PSMA-PET demonstrates superior detection of nodal and distant metastases compared with conventional imaging, supporting more accurate risk stratification and more individualized treatment planning. Its clinical impact extends beyond diagnosis: it frequently alters treatment intent, refines radiation therapy field design, informs the initiation of systemic therapy, and helps identify candidates for metastasis-directed and radioligand therapies. The incorporation of PSMA-PET into major international guidelines reflects the strength of the accumulated evidence supporting its diagnostic and clinical utility.

Important challenges remain, including interobserver variability in interpretation, false-positive findings, the absence of universal histologic validation, disparities in access, and a current lack of mature survival data linking imaging-guided management changes to long-term outcomes. The risk of stage migration without survival benefit also requires further evaluation in prospective trials. As outcome-driven research matures and reporting standards continue to develop, PSMA-PET is likely to further refine the precision of PCa care. Its ultimate value will lie not in detecting more disease, but in enabling treatment plans that align more closely with each patient's true biological disease burden, a meaningful step toward personalized, evidence-based cancer care.
